# Comparative analysis of flexural strength of abraded and polished porcelain surfaces – an in vitro study

**DOI:** 10.25122/jml-2020-0085

**Published:** 2021

**Authors:** Prathibha Nandagiri, Mamidi Praveen, Shikha Singh, Monika Singh, Namratha Chandrahari, Fayez Hussain Niazi

**Affiliations:** 1. Sparkles Dental Clinic, Lalapet, Secunderabad, Hyderabad, Telangana, India; 2. Department of Prosthodontics, Panineeya Institute of Dental Sciences, Hyderabad, Telangana, India; 3. Department of Conservative Dentistry and Endodontics, People’s Dental College of Dental Sciences and Research Center, Bhopal, India; 4. Department of Conservative Dentistry and Endodontics, Modern Dental College and Research Center, Indore, India; 5. Clove Dental Hospital, Hyderabad Telangana, India; 6. Department of Restorative and Prosthetics Dental Sciences, College of Dentistry, Dar Al Uloom University, Riyadh, Saudi Arabia

**Keywords:** flexural strength, glazed ceramics, polished ceramics, Sof-Lex finishing and polishing system, universal testing machine

## Abstract

Typically, prosthodontists adjust ceramic restorations glazed surface by grinding prior to insertion. Such alterations of surfaces are necessary for the correction of occlusal interferences. We aimed to evaluate and compare the change in flexural strength of ceramic surfaces after re-glazing and polishing. This study included 40 samples of ceramic blocks that were fabricated and glazed, and then fired in accordance with the manufacturer’s recommendations. The sample was randomly divided into four groups of 10 samples each. The first group was the control group with unaltered glazed samples. The second group was abraded with an extra-fine diamond bur followed by re-glazing, and the other two groups were polished with two commercially available polishing kits after abrading them with an extra-fine diamond bur. The samples were tested for their flexural strength using a universal testing machine. On the application of the F test on the means of all the groups, a value greater than 0.05 was found, which meant that there is no statistically significant difference in flexural strength values between the groups (P-value>0.05). Since the flexural strength values of the polished group were comparable to the other groups, polishing can be used instead of re-glazing for ceramic restorations. This reduces an additional clinical appointment for the patient and saves working time.

## Introduction

Dental ceramic has been used in dentistry for over 150 years. Currently, dental ceramic is used widely as a restorative material like all-ceramic restorations, metal ceramic crowns and fixed partial dentures because of its aesthetic properties, durability, and biocompatibility [1]. Glazing of the ceramic surface is a form of superficial treatment. It is done to seal the open pores on the ceramic surface after the firing process, thereby resulting in exceptional optical properties and superior surface smoothness. Therefore, it is vital to have an intact glazed surface of restorations to preserve its mechanical strength and also to lessen the biofilm accumulation [2].

Prior to insertion, certain surface modifications are carried out to correct occlusal interferences, contours, margins and to improve the aesthetic appearance and surface smoothness of ceramic restorations [3]. Occlusal adjustment by diamond rotary instruments causes removal of the smooth glazed surface layer and introduces surface flaws that act as focal points for crack propagation and also reduced flexural strength. These modifications compromise the quality of the restoration and have an effect on its marginal integrity and also on the oral health of soft tissues [4, 5].

To reduce the abrasiveness on the ceramic restorations created after chairside adjustments, glazing is performed again, requiring an extra clinical appointment since it is uncommon practice to have a ceramic firing oven in dental offices. To solve these problems, direct finishing and polishing are done on the restoration surface intraorally, which produces a more uniform surface. Therefore, in order to improve the flexural strength and esthetics of the restoration, glazing or polishing is carried out after the adjustment procedure [2, 3]. The purpose of this in vitro study was to assess the flexural strength between re-glazed and polished porcelain surfaces after abrasion.

## Material and Methods

The present in vitro study with a sample size of 40 was conducted to evaluate and compare the flexural strength of re-glazed and polished ceramic surfaces. Feldspathic porcelain Vita VMK Master (VITA Zahnfabrik, Bad Sackingen, Germany) was selected. A plastic mold was fabricated to achieve standardized blocks of ceramic. The mold had a length of 30 mm, a width of 6 mm, and a depth of 3 mm.

### Fabrication of test samples

Samples were fabricated using a rectangular plastic mold with perforations in the middle (30 mm length, 6 mm width, 3 mm thickness), which determined the specimen dimensions according to ISO standards (ISO6872) [6].

Each sample was fabricated by mixing porcelain powder with an adequate amount of modeling fluid. To remove excess liquid, tissue paper was placed at one end of the mass. While the mixed mass was not being allowed to dry totally, it was loaded into the mold in increments. After the complete condensation of the powder in the mold, the glass plate was used to slide over the condensed mass. The condensed mass was tenderly tapped to get released from the mold and fall over a fibrous tray support.

The fibrous tray support was placed on the firing tray, and then it was placed in the programmable furnace (Touch and Press-Dentsply) and fired according to the manufacturer’s recommendations. In a similar manner, 40 ceramic samples were prepared. Glaze (Vita AKZENT glaze powder and liquid) was applied for all the samples on one side and then fired according to the manufacturer’s recommendations. Then, the 40 samples were randomly divided into 4 groups:

• Group I (control group) – Unaltered glazed samples;
• Group II – Glazed samples abraded with an extra-fine finishing diamond bur and set for re-glazing;
• Group III – Glazed samples abraded with an extra-fine finishing diamond bur and polished with Sof-Lex finishing and polishing system;
• Group IV – Glazed samples abraded with an extra-fine finishing diamond bur and polished with a Shofu porcelain adjustment kit.

For group II samples, one side of each specimen was abraded with an extra-fine diamond rotary instrument using an air rotor handpiece to simulate clinical procedures. All the samples were abraded by placing them on the prepared acrylic block fixed on the surveyor table. The bur was applied over the specimen surface producing linear contact, and moved from left to right, in multiple stokes, to cover the block surface evenly for 10s. Then, all the samples from group II were re-glazed using the Vita AKZENT glaze powder and liquid.

**Figure 1.**
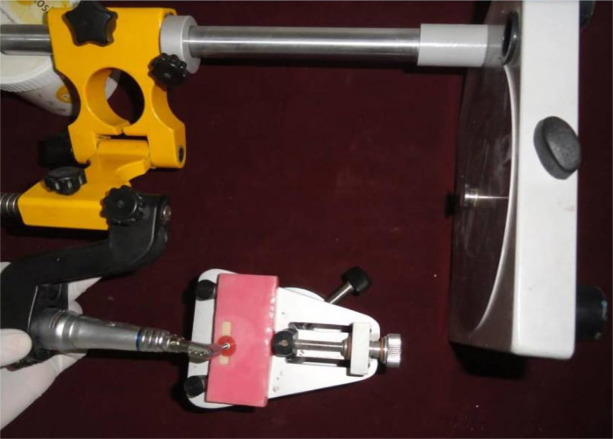
Polishing of a sample using Sof-Lex discs.

For group III samples, abrasion was done in a similar manner to group II samples, and polishing was performed using the Sof-Lex finishing and polishing system (Figure 1). A polishing sequence was then followed – from the coarse grit to super-fine discs. The polishing motion should be constant and unidirectional. The coarse and medium grit discs were used at 10,000 rpm for 15s. Fine and super-fine grit discs were then used at 30,000 rpm for 15s, each with a contra-angle handpiece according to the manufacturer’s recommendation.

**Figure 2.**
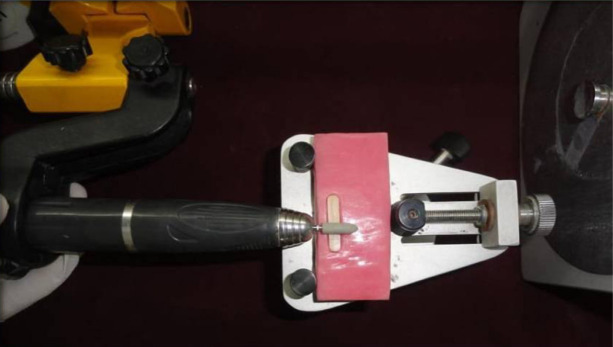
Polishing of a sample using the Shofu finishing and polishing system.

For group IV samples, abrasion was done in a similar way to group II samples, and an adjustment kit (Porcelain adjustment kit, Shofu Dental GmgH, Ratingen, Germany) was used for polishing the deglazed surfaces (Figure 2). It consisted of a sequential polishing process with three polishers of decreasing particle sizes. The Shofu abrasive rubber system sequence was composed of Ceramiste standard rubbers used for pre-polishing, Ultra for polishing, and Ultra II for high brightness polishing. All the rubbers were fitted to a micromotor handpiece calibrated at a speed of 15,000 rpm, according to the manufacturer’s recommendations.

### Testing of the samples

**Figure 3.**
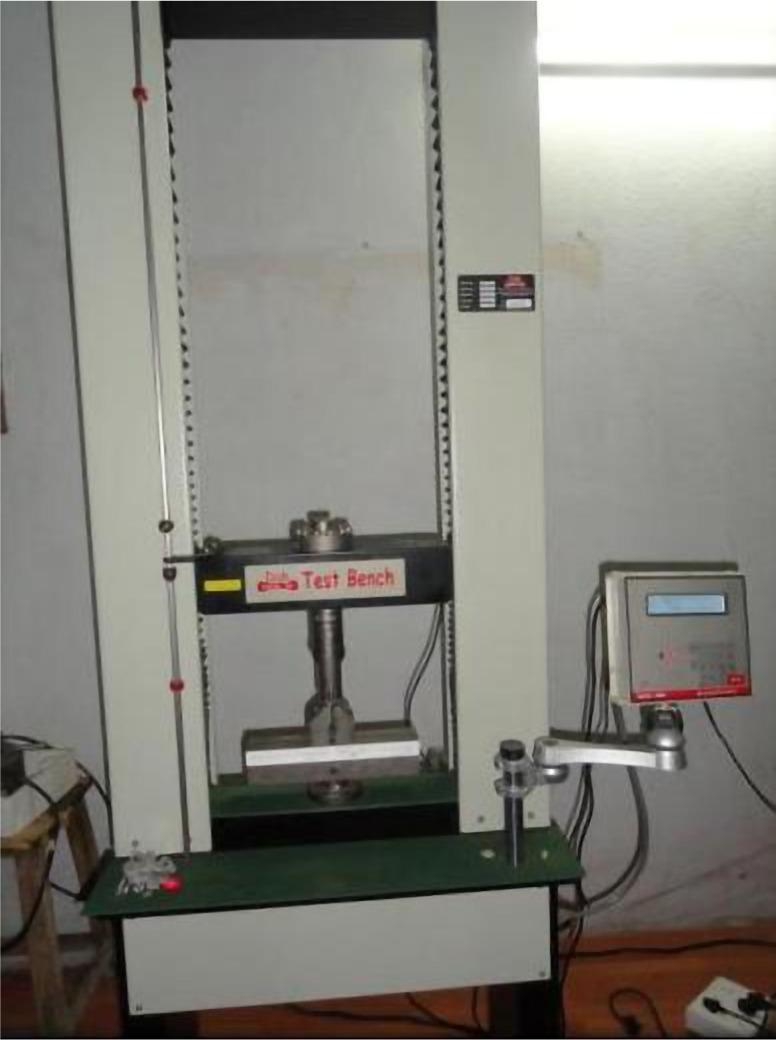
Universal testing machine.

The samples were tested for their flexural strength using a Universal Testing Machine (UTM) (Figure 3). A plunger of 1 mm cross-section with a cross-head speed of 0.5 mm/min was used. Force for fracture was recorded for each sample, and the corresponding flexural strength was calculated.

The samples were tested for their flexural strength using the three-point bend test with a UTM. Fracture force was recorded for each sample, and the flexural strength was calculated by the formula: σ = 3Fl/2xy2, where, σ denotes flexural strength; F the maximum force at the point of fracture; l implies the distance between the supports (taken as 10 mm); x is the width of the specimen, and y is the depth or thickness of the specimen. For all the samples, the peak force at the time of failure was recorded and tabulated. Using these values, the corresponding flexural strengths were calculated for each of the samples. Readings for each group were recorded in this manner, and the data for the 4 groups were collected. The observations were subjected to statistical analysis.

### Statistical Analysis

Descriptive statistics, including mean, standard deviation values, were calculated for each of the groups tested. The student’s t-test was used to analyze the significant differences between the two groups, and the F test was used to determine significant differences between control and other groups. All the analysis was done using the SPSS (Statistical Package for Social Sciences) software, version 21. A p-value of <0.05 was considered statistically significant.

## Results

The mean flexural strength of all the groups was tabulated (Table 1). The flexural strength between the control and the other groups (F test) showed a p-value greater than 0.05, which meant that there is no statistically significant difference in flexural strength values (Table 2). The student’s t-test was done to compare and analyze the flexural strength between the four study groups. A p-value >0.05 was found, indicating a statistically insignificant difference between any of the groups (Table 3).

**Table 1:** The mean flexural strength (MPa) of the four study groups.

Variable	**Group I**	**Group II**	**Group III**	**Group IV**
Mean	131.5	125.4	123.3	124.7
Standard Deviation	12.55	13.89	10.81	12.31

**Table 2:** F-test two-sample for variances.

	**Group 1 vs. Group 2**		**Group 1 vs. Group 3**		**Group 1 vs. Group 4**	
Mean	131.5	125.4	131.5	123.3	131.5	124.7
Variance	157.38	192.93	157.38	116.9	157.38	151.566
Observations	10	10	10	10	10	10
Df	9	9	9	9	9	9
F	0.81		1.34		1.03	
P (F≤f) one-tail	0.38		0.33		0.47	
F Critical one-tail	0.31		3.17		3.17	
	**Group 2 vs. Group 3**		**Group 2 vs. Group 4**		**Group 3 vs. Group 4**	
Mean	125.4	123.3	125.4	124.7	123.3	124.7
Variance	192.93	116.9	192.93	151.56	116.9	151.56
Observations	10	10	10	10	10	10
Df	9	9	9	9	9	9
F	1.65		1.27		0.771	
P (F≤f) one-tail	0.23		0.36		0.352	
F Critical one-tail	3.178		3.17		0.314	

**Table 3:** T-test: two-sample assuming equal variances.

	**Group 1 vs. Group 2**		**Group 1 vs. Group 3**		**Group 1 vs. Group 4**	
Mean	131.5	125.4	131.5	123.3	131.5	124.7
Variance	157.388888	192.933333	157.388888	116.9	157.388888	151.566666
Observations	10	10	10	10	10	10
Pooled Variance	175.161111		137.144444		154.477777	
Hypothesized Mean Difference	0		0		0	
Df	18		18		18	
t Stat	1.03061389		1.56570430		1.22337868	
P (T≤t) one-tail	0.15818605		0.06741439		0.11848070	
t Critical one-tail	1.73406360		1.73406360		1.73406360	
P (T≤t) two-tail	0.31637211		0.13482879		0.23696140	
t Critical two-tail	2.10092204		2.10092204		2.10092204	
	**Group 2 vs. Group 3**		**Group 2 vs. Group 4**		**Group 3 vs. Group 4**	
Mean	125.4	123.3	125.4	124.7	123.3	124.7
Variance	192.933333	116.9	192.933333	151.566666	116.9	151.566666
Observations	10	10	10	10	10	10
Pooled Variance	154.916666		172.25		134.233333	
Hypothesized Mean Difference	0		0		0	
Df	18		18		18	
t Stat	0.37727256		0.11926236		0.27019844	
P (Tt) one-tail	0.35519118		0.45319423		0.39504058	
t Critical one-tail	1.73406360		1.73406360		1.73406360	
P (T≤t) two-tail	0.71038236		0.90638847		0.79008117	
t Critical two-tail	2.10092204		2.10092204		2.10092204	

Analysis of the statistical data revealed no statistically significant difference between the control and the re-glazed samples (group I and group II), although the mean values for group I were greater. There was no statistically significant difference between any of the groups. This shows that the flexural strength of abraded and re-glazed group (group II) was comparable to that of the polished groups (group III and group IV).

The mean value for glazed samples was higher than the polish groups. On the application of the F test on the means, a P-value of 0.2 (>0.05) was found, which is not statistically significant. In the present study, a P-value of 0.3 was found, which shows that there is no significant difference in the flexural strength values of both the polished groups. Consequently, it can be concluded that either of the polishing systems i.e., Sof-Lex finishing and polishing system or Shofu porcelain adjustment kit, can be used for polishing the ceramic surfaces.

## Discussion

Several methods such as shot peening, transformation saturation, dispersion strengthening of glasses, strengthen with a metal substructure, controlled crystallization of glasses, enameling of high strength crystalline ceramics, production of prestressed surface layers in dental porcelain via ion exchange, thermal tempering, crack tip blunting and minimizing the number of firing cycles are used to strengthen dental porcelains. While oven glazed porcelain is acknowledged as the gold standard to obtain the best polishing characteristics, nowadays, many polishing kits are obtainable in the market for this purpose [7]. 

The main benefits of effective finishing and polishing are aesthetics, marginal integrity, and oral health of soft tissues. However, finishing and polishing procedures produce wear of dental restorative material surfaces. The objective of glazing is to seal the surface of fired porcelain, thereby effectively reducing the crack propagation [8].

Several authors supported the usage of polishing as an alternative for glazing. Rosenstiel *et al.* found greater fracture toughness of polished porcelain than that of glazed porcelain [9]. Albakry *et al.* concluded that different surface treatments do not affect the strength of the material. Ahmad *et al.* carried a scanning electron microscopy (SEM) analysis of flexural strength and surface smoothness of aluminous dental ceramic material and found that polishing with high polishing speed (20,000 rpm) diamond burs did reduce the strength of the material, and auto glazing did not cause any improvement in flexural strength [11]. 

Jagger *et al.* used Sof-Lex and Shofu polishing kits to polish the porcelain groups and found reduced enamel wear in the polished porcelain groups. On the other hand, a statistically insignificant difference was seen between the glazed and the unglazed porcelain groups [12].

Olivera *et al.* found that polished ceramics showed a greater degree of roughness when compared to their glazed counterparts. According to the authors, the microstructural differences between the different ceramic materials may be more important than their superficial roughness and concluded that usage of Shofu kit for polishing and finishing is beneficial [13].

Gauri Mulay *et al.* concluded that the enamel wear produced by polished porcelain is substantially weaker than auto glazed and overglazed porcelain [14]. Haywood *et al.* found no difference between smoothness of veneer porcelains finished with glazing and polishing [15].

Goldstein *et al.* found that Brassier, Dedeco, Dentsply, and Shofu porcelain polishing systems were clinically acceptable for finishing. The Brassier system was superior to Ceremco porcelain, and the Den-Mat system was found unacceptable [16]. Wright *et al.* found that three polishing kits (Axis Dental, Jelenko, and Brassier) provided smoother surfaces than the auto glazed group for the polishing of ultra-low fusing dental porcelain [17].

Prosthodontists usually make occlusal adjustments before final cementation, removing the surface glaze and introducing microscopic surface flaws that may lead to chipping of the porcelain layer from the metal sub-structure, surface cracks, crack propagation, resulting in failure of the restoration. Refiring these restorations before the final placement produces a self glaze layer, which increases the strength since it decreases flaw depth and sharpness. The various disadvantages of re-glazing are marginal distortion, reduced fracture toughness, and an extra appointment for the patient [1, 5].

In the present in vitro study, the flexural strength of re-glazed, finished, and polished ceramic surfaces has been evaluated. Here, ceramic surfaces were abraded by extra-fine diamond bur. Shofu porcelain adjustment kit and Sof-Lex finishing and polishing system were used to polish the feldspathic dental porcelain surface after abrading it with an extra-fine diamond rotary instrument.

Analysis of the statistical data revealed no statistically significant difference between the control and the re-glazed samples (group I and group II), although the mean values were greater in group I. There was no statistically significant difference between any of the groups. This shows that the flexural strength of abraded and re-glazed group (group II) was comparable to that of the polished groups (group III and group IV). The mean value was higher for glazed samples compared to the polish groups. Therefore, it is suggested that a direct finishing and polishing procedure on the restoration surface can be done intraorally with the commercially available polishing kits as an alternative procedure to re-glazing. 

## Conclusion

The chairside polishing of the ceramic surfaces using the polishing kits could be a better alternative clinical step, especially in the post-cementation stage where the clinician requires the adjustment of the restoration. We found that either of the polishing systems, i.e., Sof-Lex finishing and polishing system or Shofu porcelain adjustment kit, can be used for polishing the ceramic surfaces. Further studies should be undertaken to establish these findings.

## Acknowledgments

### Conflict of interest

The authors declare that there is no conflict of interest.
